# Flunarizine Versus Propranolol in Prophylaxis of Pediatric Migraine: An Open-Label Randomized Trial

**DOI:** 10.7759/cureus.74563

**Published:** 2024-11-27

**Authors:** ND Vaswani, Preet Lamba, Vandana Arya, Seema Lekhwani

**Affiliations:** 1 Pediatrics, Pandit Bhagwat Dayal Sharma Post Graduate Institute of Medical Sciences, Rohtak, IND; 2 Pediatrics, Government Medical College & Hospital, Chandigarh, IND; 3 Biochemistry, Pandit Bhagwat Dayal Sharma Post Graduate Institute of Medical Sciences, Rohtak, IND

**Keywords:** children, flunarizine, pediatric migraine, pedmidas, propranolol

## Abstract

Background

Pediatric migraine is a primary headache affecting daily activities and causing significant disability among children. However, clarity on the usage of prophylactic medications in children is yet to be established. This study was conducted with the aim of comparing the efficacy and safety of flunarizine and propranolol in the prophylaxis of pediatric migraine.

Methodology

An open-label randomized trial with parallel group assignment was conducted in the Department of Pediatrics of a tertiary care hospital in Northern India among patients aged five to 14 years with migraine having Pediatric Migraine Disability Assessment (PedMIDAS) score of 11 to 139 and a headache frequency of four or more days over a baseline period of 28 days. Enrolled patients were assigned randomly to receive either flunarizine (5 mg/day HS for the first month and then 10 mg/day HS for the next two months) or propranolol (1 mg/kg/day in two divided doses for three months) and then followed up monthly for three months for outcomes. The primary outcome was the proportion of children with a 50% or more reduction in the number of headache days compared to the 28-day baseline period with the last 28 days of the 12-week trial period. Secondary outcomes were headache-related disability (as measured by the absolute change in PedMIDAS score), the absolute change in the number of headache days, and the proportion and nature of adverse effects in the two groups.

Results

A total of 40 patients underwent randomization (20 in each group). Baseline parameters were comparable in the two groups. The primary outcome, that is, a 50% or more reduction in the number of headache days, was achieved in 10 out of 20 (50%) patients in the flunarizine group and 11 out of 20 (55%) patients in the propranolol group (p = 0.752). Both groups were comparable in terms of the primary outcome. There were also no significant between-group differences in terms of headache-related disability (change in PedMIDAS: 8.2 ± 2.97 in the flunarizine group vs 8.7 ± 3.95 in the propranolol group, p = 0.924) and absolute reduction in the number of headache days (4.3 ± 2.36 in the flunarizine group vs 4.3 ± 2.11 in the propranolol group, p = 0.989). Minor adverse effects like nausea, vomiting, drowsiness, and fatigue were comparable in the two groups. None of the patients reported any serious adverse events.

Conclusion

Flunarizine is as effective as propranolol for the prophylactic management of children with migraine. Both drugs were well-tolerated and safe.

## Introduction

The word “migraine” comes from the Greek word “hemicrania,” meaning half of the head. The Headache Classification Committee of the International Headache Society (ICHD-3, 2018) defines migraine as episodic attacks of headache lasting four to 72 hours with two of the following symptoms: unilateral pain, throbbing, pain of moderate or severe intensity, or aggravation on movement, and one of the following symptoms: photophobia or phonophobia, nausea, or vomiting [1]. The International Headache Society criteria for diagnosing pediatric migraine have evolved over time, with revisions aimed at capturing the unique clinical features and characteristics of migraine in children and adolescents, including headaches of bilateral location and also those of shorter duration (two hours).

The prevalence of migraines in the pediatric population ranges from 10% to 40%, which rises with age [2,3]. Migraine occurs at all ages and may even begin in infancy [4]. It has been seen that up to 18% of patients in the pediatric emergency department had migraine-related symptoms. Headache frequency and intensity contribute to significant headache-related disability, and children with migraine experience a lower quality of life and have impairments in their school performance [5-7]. Thus, migraine is a major burden on daily quality of life and requires appropriate and timely treatment.

Migraines in the adult and pediatric populations are substantially different clinical entities, and one cannot assume that a drug effective in adults will also be effective in children. Currently, there is insufficient evidence on the treatment of pediatric migraine, and most of the management has relied on extrapolation data from adult studies. Therefore, the U.S. Food and Drug Administration (FDA) has emphasized the need for more studies on the pediatric population [8]. The role of flunarizine has been studied in several randomized trials conducted among adults [9-11] as well as children [12,13] and has been shown to result in significant headache reduction. Efficacy trials of propranolol in the prevention of migraine headaches have also shown variable results [14,15].

Landmark studies such as the Childhood and Adolescent Migraine Prevention (CHAMP) trial, conducted by Powers et al., have played a pivotal role in advancing our understanding of effective preventive strategies for managing pediatric migraine [16]. The findings of the CHAMP trial have significant implications for clinical practice, emphasizing the importance of early intervention and individualized treatment approaches in pediatric migraine management [16].

Considering the substantial lack of studies on the comparison of the clinical efficacy of propranolol and flunarizine in the pediatric population, the present study was designed with the aim to compare the efficacy and safety of the two drugs in prophylaxis of pediatric migraine.

## Materials and methods

This open-label randomized trial with parallel group assignment was conducted in the Department of Pediatrics of a tertiary care hospital in Northern India. Patient enrollment was commenced after obtaining approval from the institutional ethics committee (IEC approval no. BREC/Th/20/007). The trial was registered under Clinical Trials Registry of India (CTRI) No. CTRI/2021/12/039008. Study participants were recruited from the pediatric outpatient department. Children aged five to 14 years diagnosed with migraine were assessed for eligibility criteria. These children were subjected to a baseline period of 28 days, during which no prophylactic therapy was administered, and only symptomatic treatment was continued. During this period, patients were advised to maintain a headache diary that contained information on the date and time of headache episodes, their intensity and duration in hours, and the use of analgesic drugs and their side effects, if any. Children scoring between 11 and 139 on the Pediatric Migraine Disability Assessment (PedMIDAS) score and experiencing a headache frequency of four or more days over the 28-day baseline period were eligible for inclusion. Exclusion criteria included the presence of chronic diseases such as epilepsy, chronic liver disease, chronic kidney disease, hypertension (blood pressure >95th percentile for age, sex, and height; or >130/80 mm Hg for age >13 years), diabetes (fasting plasma glucose >126 mg/dL or two-hour plasma glucose during oral glucose tolerance test >200 mg/dL), and obesity (body mass index >95th percentile for age and sex).

Informed and written consent from the parents or guardians of all eligible participants, and when appropriate, child assent was obtained. Qualified participants were randomized into two groups using computer-generated variable size and block randomization (two, four, or six) sequence, and allocation concealment was done using the sealed envelope technique. Considering the lack of data on the efficacy of both drugs as well as the time constraints and other local logistics, a convenience sample of 20 in each group was taken. Group A children received tablet flunarizine in the dose of 5 mg/day HS for the first month and then 10 mg/day HS for the next two months. A lower dose of 5 mg was started to improve patient tolerance, particularly for common side effects like tiredness and drowsiness. Group B children received tablet propranolol 1 mg/kg/day in two divided doses for three months. During the study period of three months, patients were asked to continue maintaining the headache diary and to report all adverse events, with follow-up planned at the end of each month. PedMIDAS scores were recalculated at the end of the study period. A comparison was made between the 28-day baseline period and the last 28 days of the 12-week trial.

The primary outcome was the proportion of children with a 50% or more reduction in the number of headache days on comparing the 28-day baseline period with the last 28 days of the 12-week trial period. Secondary outcomes were headache disability (as measured by the absolute change in PedMIDAS score), the absolute change in the number of headache days, and the proportion and nature of adverse effects in the two groups.

The data entry was done in the Microsoft Excel spreadsheet, and the final analysis was done with the use of IBM SPSS Statistics for Windows, version 25.0 (IBM Corp., Armonk, NY). The presentation of categorical variables was done in the form of numbers and percentages (%). On the other hand, the quantitative data were presented as mean ± SD or as median with 25th and 75th percentiles (interquartile range). The data normality was checked using the Shapiro-Wilk test. The quantitative variables were analyzed using the independent t-test, and the qualitative variables were analyzed using the chi-square test or Fisher’s exact test. For statistical significance, a p-value of less than 0.05 was considered statistically significant.

## Results

**Figure 1:**
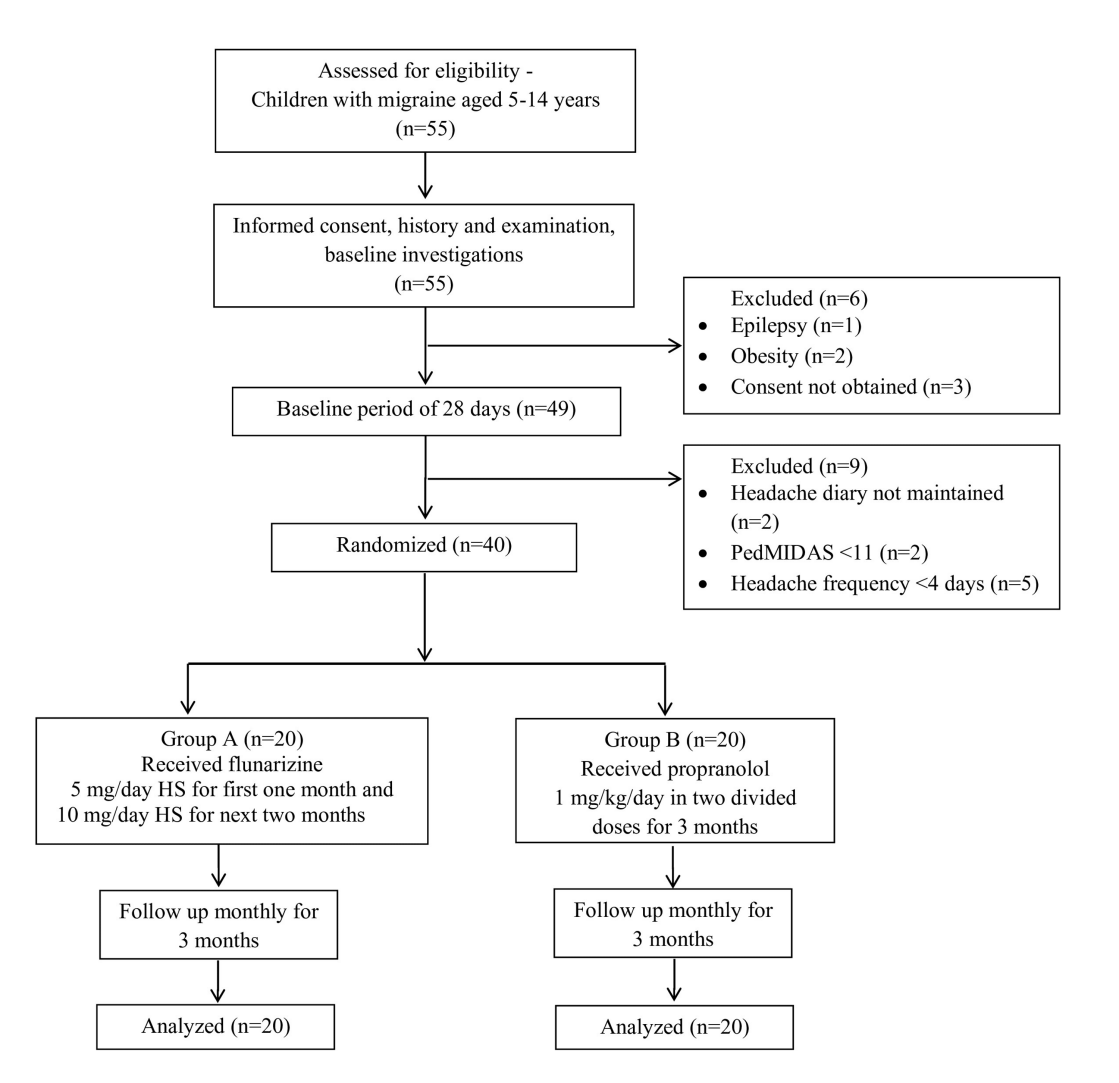
Study flow chart

A total of 55 children and adolescents were assessed for eligibility, out of which 40 underwent randomization to receive either flunarizine (20 patients) or propranolol (20 patients) (Figure 1). The baseline demographic variables were comparable between the two groups in terms of age (p = 0.367) and gender (p = 1). The mean (±SD) age of all patients was 9.9 ± 2.07 years, and 55% of all were females. The mean number of headache days during the 28-day baseline period diary recordings for all patients was 9 ± 2.75, and the mean baseline PedMIDAS score was 30.52 ± 7.83. The clinical characteristics of the headache were also comparable between both groups in terms of location of pain (p = 0.525), lateralization of headache (p = 0.519), duration of episodes (p = 0.805), and frequency of episodes (p = 0.48). The number of headache days during the 28-day baseline period and PedMIDAS score were also similar in the two study groups (Table 1).

**Table 1:** Comparison of the baseline characteristics of the patients in the two arms

Characteristic	Flunarizine (n = 20)	Propranolol (n = 20)	p-value
Age (years)	10.2 ± 2.21	9.6 ± 1.93	0.367
Female gender	11 (55%)	11 (55%)	1
Location of headache			
Frontal	10 (50%)	12 (60%)	0.525
Temporal	10 (50%)	8 (40%)	
Unilateral headache	7 (35%)	9 (45%)	0.519
Duration of episode			
<6 hours	10 (50%)	8 (40%)	0.805
6 to 12 hours	9 (45%)	10 (50%)	
>12 hours	1 (5%)	2 (10%)	
Frequency of episodes			
<3/week	16 (80%)	13 (65%)	0.48
>3/week	4 (20%)	7 (35%)	
Family history of migraine	6 (30%)	3 (15%)	0.451
No. of headache days during the 28-day baseline period	9.15 ± 2.62	8.85 ± 2.92	0.735
PedMIDAS score	31.05 ± 6.97	30 ± 8.76	0.677

On comparing the 28-day baseline period with the last 28 days of the 12-week trial period, the primary outcome measure, that is, a 50% or more reduction in the number of headache days, was achieved in 10 out of 20 (50%) patients in the flunarizine group and 11 out of 20 (55%) patients in the propranolol group (p = 0.752). The efficacy of flunarizine and propranolol were comparable in terms of a 50% or more reduction in headache days. Also, there were no significant between-group differences in terms of the secondary outcome measures, that is, headache-related disability (absolute change in PedMIDAS score: 8.2 ± 2.97 in the flunarizine group vs 8.7 ± 3.95 in the propranolol group, p = 0.924) and absolute reduction in a number of headache days (4.3 ± 2.36 in the flunarizine group vs 4.3 ± 2.11 in the propranolol group, p = 0.989) (Table 2). Minor adverse effects that occurred in the flunarizine and propranolol groups were nausea and vomiting (5% vs 20%) and drowsiness and fatigue (40% vs 15%) and were comparable in the two groups. None of the patients reported any serious adverse events.

**Table 2:** Comparison of the primary and secondary outcome variables in the two arms

Outcome	Flunarizine (n = 20)	Propranolol (n = 20)	p-value
Primary outcome			
≥50% relative reduction in headache frequency no. (%)	10 (50%)	11 (55%)	0.752
Secondary outcome			
Change in PedMIDAS score	8.2 ± 2.97	8.7 ± 3.95	0.924
An absolute reduction in headache days	4.3 ± 2.36	4.3 ± 2.11	0.989
Adverse effects			
Nausea and vomiting	1 (5%)	4 (20%)	0.114
Drowsiness and fatigue	8 (40%)	3 (15%)	0.342

## Discussion

Our study demonstrated that flunarizine and propranolol were equally efficacious prophylactic medications for migraine in children. The primary outcome of the present study was the proportion of patients with at least a 50% reduction in headache days, which was found to be comparable between flunarizine and propranolol (50% and 55%, respectively) (p = 0.752). The absolute reduction in the number of headache days and the absolute change in PedMIDAS score were also similar with both flunarizine and propranolol.

Several classes of medications are used for preventive therapy for migraine and include tricyclic antidepressants; antiepileptics like valproate sodium, topiramate (TPM), and levetiracetam; calcium channel blockers like flunarizine; and antihypertensives like propranolol. The decision to start a preventive medication requires careful consideration of potential risks and benefits. The efficacy and safety of flunarizine have been studied in the pediatric population. In a retrospective observational audit conducted by Mohamed et al., a 50% reduction in attack frequency was observed in 57% (41/72) of children with flunarizine [17]. Visudtibhan et al. also found that nine patients out of 21 children receiving flunarizine (42%) had more than a 50% reduction in the frequency of migraine [12]. A similar efficacy of flunarizine in terms of a 50% or more reduction in headache days was also observed in our study.

Randomized trials comparing the efficacy and safety of flunarizine and propranolol have been conducted in the adult population, showing no significant difference between the two drugs [9,18]. Raybarman studied the efficacy of combined low doses of propranolol (20 mg) and flunarizine (5 mg) in episodic migraine and suggested its use in patients who don’t respond to flunarizine monotherapy [19].

In the index study, disability caused by migraine was also assessed in terms of PedMIDAS score, which was assessed at the beginning of the study. The scores were compared between the two groups, and no significant difference between the propranolol and flunarizine groups was seen after the prospective baseline period of 28 days (p = 0.677). The PedMIDAS scores were recorded monthly and compared between two groups, later found to be statistically comparable [after the first month of medication (p = 0.85), after the second month of medication (p = 0.635), and after the third month of medication (p = 0.582)]. Bhat et al. also observed that the mean MIDAS scores for any of the therapy groups (propranolol, flunarizine, and divalproex sodium) were not significantly different (p = 0.721) [18]. On the other hand, when the final MIDAS scores were compared to the ones at the beginning of the treatment period, it was discovered that all of the treatment groups saw a statistically significant improvement (p = 0.001). The differences between the groups were not statistically significant (p = 0.766). Though conducted in adults, the study by Bhat et al. exhibits similar outcomes to our study [18].

Studies evaluating the role of propranolol in prophylaxis of migraine have shown variable results. Dakhale et al. reported that the percentage of responders (i.e., >50% decrease in mean headache frequency) at the end of 12 weeks and a decrease in mean headache frequency was better in propranolol when compared to low-dose sodium valproate [20]. On the other hand, a study done to compare the efficacy and safety of TPM and propranolol for migraine prophylaxis in children concluded that TPM is more effective than propranolol for pediatric migraine prophylaxis [14].

In the current study, no serious adverse effects were noted in either group. None of them required discontinuation of treatment. Drowsiness and fatigue were reported in 40% of patients receiving flunarizine, although these side effects were not found to interfere with daily routine activities. A previous study comparing flunarizine and propranolol has reported that propranolol significantly reduced blood pressure and heart rate; flunarizine had no effect on cardiovascular function, and weight gain was noted with both treatments. They concluded that flunarizine may have a better safety profile [9]. No cardiovascular side effects were observed with the use of propranolol in our study. Also, no weight gain was noted in our study, which can be attributed to the small sample size and duration of the study.

The CHAMP trial, a multicenter, randomized, double-blind, placebo-controlled trial, evaluated the efficacy of preventive medications in children and adolescents with migraine. A diverse cohort of pediatric migraine patients were enrolled and randomized to receive either amitriptyline, TPM, or placebo over a 24-week period. The study demonstrated that there were no significant differences in reduction in headache frequency or headache-related disability with amitriptyline, TPM, or placebo over a period of 24 weeks. The active drugs were associated with higher rates of adverse effects. This trial emphasized the role of high placebo response rates in pediatric migraine. Not using a placebo as a control is a limitation of our study. Other limitations include the small sample size and the inability to follow-up for longer periods. Further studies involving larger sample sizes and longer follow-up periods are required for the strengthening of the evidence.

## Conclusions

We concluded that the drug flunarizine is as effective as propranolol for the prophylactic management of children with migraine. When compared to the initial condition, the disability brought on by migraine headaches was reduced as a result of the effects of both medicines. Both drugs were well-tolerated and safe.
